# Short term-outcomes of periacetabular osteotomy in the neuromuscular hip

**DOI:** 10.1093/jhps/hnaf050

**Published:** 2025-08-25

**Authors:** Tobias Stedman, Scott Ryan Booth, Nick Green, Caroline Blakey, Sanjeev Madan

**Affiliations:** Department of Trauma and Orthopaedics, Sheffield Children’s Hospital, Western Bank, Sheffield S10 2TH, United Kingdom; Sheffield Medical School, University of Sheffield, Beech Hill Rd, Broomhall, Sheffield S10 2RX, United Kingdom; Department of Trauma and Orthopaedics, Sheffield Children’s Hospital, Western Bank, Sheffield S10 2TH, United Kingdom; Department of Trauma and Orthopaedics, Sheffield Children’s Hospital, Western Bank, Sheffield S10 2TH, United Kingdom; Department of Trauma and Orthopaedics, Doncaster Royal infirmary Thorne Rd, Doncaster DN2 5LT, United Kingdom; Department of Trauma and Orthopaedics, Sheffield Children’s Hospital, Western Bank, Sheffield S10 2TH, United Kingdom; Department of Trauma and Orthopaedics, Doncaster Royal infirmary Thorne Rd, Doncaster DN2 5LT, United Kingdom

**Keywords:** peri-acetabular osteotomy, hip dysplasia, cerebral palsy, neuromuscular

## Abstract

The study aims to evaluate our experience of the Bernese periacetabular osteotomy (PAO) in patients with neurogenic hip dysplasia. Retrospective data were collected for patients with neurogenic hip dysplasia, managed with PAO over a 10-year period. Data were collected for 20 consecutive patients (23 hips). Mean age at the time of surgery was 15 years (range 12–31). 85% had a diagnosis of cerebral palsy (GMFCS 1(18%), 2(18%), 3(35%), 4(12%), 5(18%)). Mean follow up was 47 months. Electronic records were reviewed for outcomes. Pre- and post-operative radiographs were evaluated separately by two independent assessors using TraumaCad software. Post-operative radiographic evaluation was available for 20 patients (21 hips). The median lateral centre edge angle was corrected from −1° pre-operatively (range: −58° to 25°) to 42° post operatively (range: 15° to 65°). Tönnis angle was corrected from an average of 26° pre-operatively (range: 10° to 37°) to 9° post-operatively (range: −19 to 29). Reimer’s index decreased from an average of 50% (range: 27% to 93%) to 7% (range 0% to 25%) post-operatively. At the latest follow there was absence of pain in 16 patients. Our experience has demonstrated safe use of the PAO, in conjunction with soft tissue procedures and femoral osteotomy, in the management of neuromuscular patients. In this cohort there was stable reduction of the hip in all but two patients (three hips), and effective pain management for this challenging patient group.

## INTRODUCTION

Neurogenic hip dysplasia is a complex problem. Muscle imbalance and loss of normal physiological loading, with secondary bony deformities, lead to progressive hip subluxation and risk dislocation [1]. Progressive hip instability can impair mobility, compromise standing transfers and in more severely affected children cause difficulties with dressing and nursing care. A significant decrease in quality of life is seen for both patients and carers when the hips of children with neuromuscular disease subluxate or dislocate [2, 3]. Difficulties with perineal care are reported in up to 38% patients with untreated spastic hip dislocations [4–6]. Secondary issues with scoliosis and pelvic obliquity can develop, contributing to poor sitting balance and necessitating wheelchair modifications [6]. Surgical reconstruction has been shown to improve the quality of life of these patients [5, 7]. Identifying early subluxation in this patient group based on clinical assessment and range of motion alone is challenging and has been shown to be a poor indicator of at-risk hips [8]. Several countries have developed hip surveillance programmes using serial assessment and pelvic radiographs to enable early identification, and treatment, of hip dysplasia in the group of neuromuscular patients with cerebral palsy. These programmes have been shown to decrease the need for reconstructive hip surgery by enabling early soft tissue intervention, successfully preventing progression to hip dislocation [9–14]. Where bony surgery is needed, a one stage hip reconstruction has been shown to reduce the frequency and intensity of pain and improve femoral head shape [15, 16].

Despite these programmes, patients continue to present with symptomatic neuromuscular-associated hip dysplasia during adolescence or early adulthood. Reconstructive correction of neuromuscular hip dysplasia in these patients remains complex. This study aims to evaluate the experience of a UK tertiary specialist paediatric orthopaedic unit of the Bernese periacetabular osteotomy (PAO) in patients with neurogenic hip dysplasia, to provide information on outcomes for this challenging patient group.

## METHODS

Patients with a neuromuscular disorder who had undergone PAO were identified through searching a local database, theatre management systems, and clinical coding. Retrospective review of case notes and analysis of the available radiographs was performed. Pre and post-operative radiographs were assessed by two separate assessors using TraumaCad ® orthopaedic digital software (Brainlab LTD.). Radiographic analysis included measurement for lateral centre edge angle (L-CEA), Sharp’s angle, Reimer’s migration index, and Tönnis angle. The adequacy of the radiographs was ascertained using the obturator index to assess pelvic obliquity [17]. Inter-observor reliability was measured using correlation coefficients.

Demographic information included age at time of surgery, pre-operative diagnosis and previous surgery on the pelvis or femur. Length of stay, including any High Dependency Unit (HDU) admission, and estimated blood loss were documented. Complications including metalwork failure, iatrogenic fracture, post-operative infection or wound break down, need for further surgery, persistent pain, non-union, or Avascular Necrosis (AVN) were collected. All complications were graded according to the modified Calvien-Dindo-Sink classification system. The modified Calvien-Dindo-Sink classification system grades complications from I to V based on increasing severity and has been shown to have good to excellent inter-rater reliability in paediatric orthopaedic and hip preservation surgery [18, 19]. All operations were performed by the three senior authors (C.B., R.N.G., and S.M.) at a tertiary referral specialist children’s hospital in the UK. Excluded from data analysis were any patients who had less than 12 months follow up.

### Surgical technique

In our practice, children and young people with neuromuscular conditions are referred for consideration of reconstruction in the presence of hip dysplasia to prevent progression of disease, to treat pain and improve function. Hip stability can improve gait and standing transfers, improved movement aiding sitting balance or help facilitate hygiene care. Indications for PAO over acetabuloplasty, in our practice, include a closed triradiate cartilage, and hip dysplasia with pain, progressive migration or instability, subluxation or dislocation. Concordant femoral osteotomies are considered in patients where it is expected that significant shortening would be required to allow de-tensioning of the soft tissues allowing for reduction of the hip and mobilization of the acetabular fragment, or in the presence of excessive torsional abnormalities. Many of these patients have developed secondary deformity of the femoral head. Incongruency is not a contra-indication to reconstruction in these patients but in this scenario reconstruction must be considered a salvage procedure. All patients considered for surgery were discussed in a regional MDT prior to listing and go through a rigorous pre-operative assessment with our anaesthetic colleagues to ensure their fitness for surgery, and to optimise where possible. Ambulant patients are assessed pre- and post-operatively at our regional gait lab where detailed recommendations regarding correction of torsional profile or additional soft tissue procedures may be made.

Where possible, pre-operative computed tomography is performed to allow three-dimensional assessment of the femoral deformity, acetabular dysplasia and torsional profile of the lower limb. This may necessitate a further general anaesthetic and timing of a hip joint injection under the same GA is useful to confirm the presence of intra-articular pain.

The femoral osteotomy is performed first in the majority of cases. We have found that in neurogenic dysplasia significant shortening is usually required to allow adequate soft tissue de-tensioning, hip reduction and acetabular fragment mobility. We aim to bring the neck shaft angle to no lower than 125°, particularly in ambulant patients. A closing trapezoidal wedge intertrochanteric osteotomy is performed, allowing psoas to be released with excision of the lesser trochanter during shortening. The amount of shortening of the femur is variable (estimated 2-3 cm) but we have found that too conservative can lead to technical difficulties with the acetabular procedure. A blade plate is used for fixation, and temporarily fixed with two screws until PAO correction, to allow modification if required for stability. De-rotation is planned to optimise the McKibbin instability index [20], reflecting the combined femoral and acetabular version. Whilst we aim for physiological femoral version, the deformity in neuromuscular hips is often complex, with secondary retroflexion of the head and needs to be carefully considered [21].

PAO is performed through a bikini incision using a minimally invasive technique. The senior author uses a trans-sartorial approach [22], while other consultant surgeons approach rectus through the bed of Tensor Fasciae Latae (TFL) and detach sartorious subperiosteally from the Anterior Superior Iliac Spine (ASIS) for later trans-osseous suture repair. The straight head of rectus is left attached at the Anterior Inferior Iliac Spine (AIIS). Subperiosteal dissection allows exposure of the superior ramus beyond the pectineal eminence, and the posterior column. The outer table musculature is left intact, other than a small lateral window at ASIS level with a curved retractor to protect the abductors during division of the ilium. A sharp Hohmans retractor in the superior ramus, retracts iliopsoas and the femoral neurovascular bundle and the osteotomies are performed in order. The ischium is approached through the interval between psoas and the hip capsule and partially osteotomised, the ramus is then osteotomised completely. A saw blade is used to divide the ilium to just short of the pelvic brim and finally the medial and lateral posterior column are osteotomised. Under image guidance, utilizing the false profile view, the medial cortex of the posterior column is scored, connecting with the ischial cut, before carefully following the osteotomy down the lateral side of the posterior column. Non-weight bearing, nutritional issues and low physical activity in these patients impacts bone health. It is not uncommon for osteotomies to be difficult, behaving like greenstick fractures. In these patients we are cautious to fully complete the medial and lateral posterior column cuts carefully to the ischium, before attempted mobilization.

Correction of the acetabular fragment is performed with the aim of achieving optimal stability rather than restoring normative radiographic parameters. These patients have significant subluxation or frank dislocation, and lower functional requirements as compared with patients with non-neurogenic hip dysplasia. Hence the risk of under-correction and ongoing instability is higher than the risk of overcorrection and impingement. Mobilization of the fragment can be difficult. With soft bone the surgeon must be cautious not lever on the Schanz pin, inserted into the supraacetabular region to aid positioning of the fragment. The pubis can elevated with a tenaculum forceps, or bone hook inserted retrograde into the canal of the superior ramus. Fixation is routinely with three large fragment screws but sometimes additional screws or recon plates are considered.

**Chart 1:**
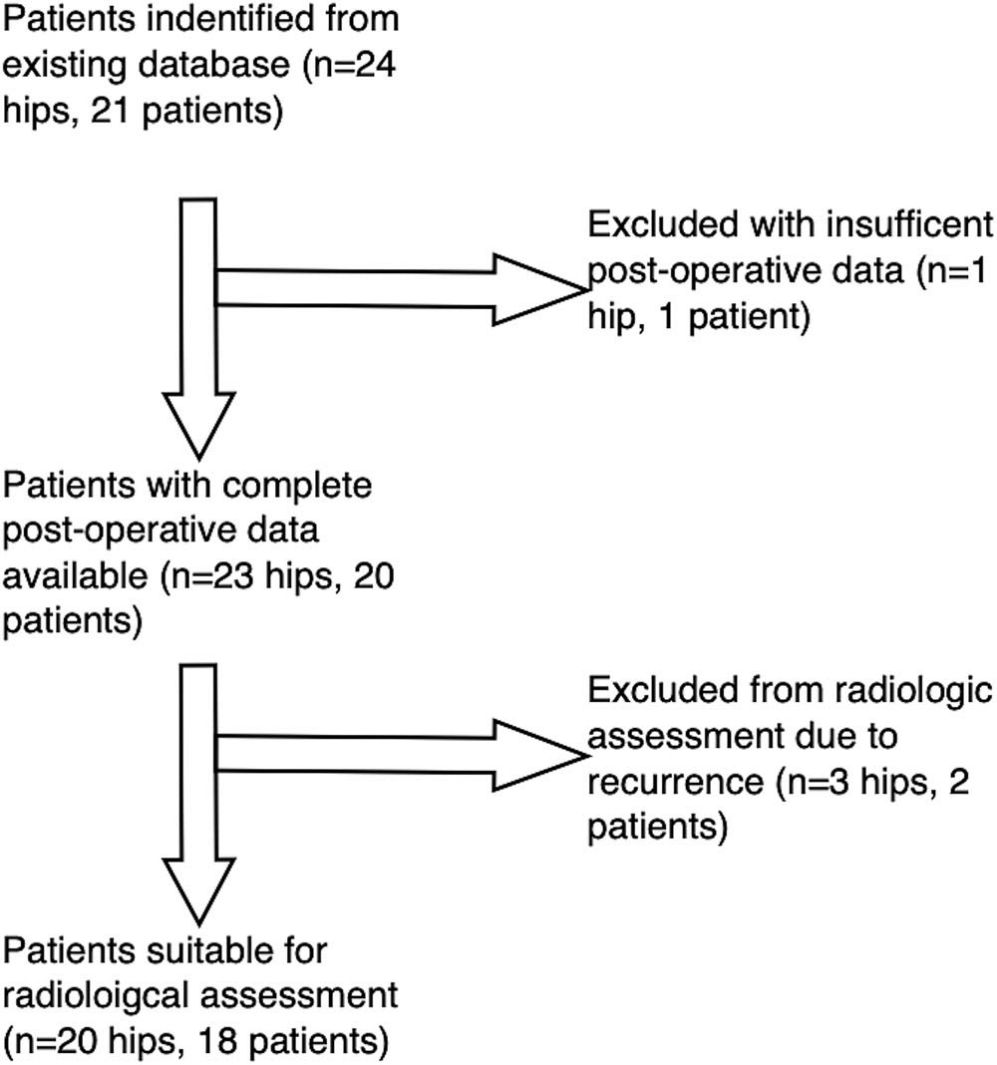
Flow chart of patient identification and selection for inclusion.

Cell salvage and tranexamic acid is routine. The leg is positioned to optimise the distance of the sciatic nerve to the osteotomies of the ischium and posterior column. Patients are given antibiotics and thromboprophylaxis. Radiographs are performed before discharge to ensure no displacement of the fragment and then at 6 weekly intervals until bony union. Whilst some authors advocate for the use of protective abduction splints [1] our practice was to not use any post operative splints or casting. Patients, where able, were allowed to touch weight bear with assistive devices under the guidance of physiotherapists for the first 6 post-operative weeks before gradually transitioning to full weight bearing.

## RESULTS

Twenty-one patients were identified, having undergone PAO in the presence of neurogenic dysplasia, over a period of 10 years (May 2013 to October 2022). Full patient demographics and diagnoses are detailed in Table 2. The average age at the time of surgery was 15 years (range 12–32). There was equal distribution of male and female patients (10 male and 11 female). About 85% (18 patients) had a diagnosis of cerebral palsy with average GMFCS 3. Three patients had bilateral surgery in separate sittings. One patient was excluded from data analysis due to insufficient post-operative data available, leaving 20 patients (23 hips). Two patients (3 hips;5, 6 and 8) had recurrence and therefore could not be included in post-operative radiological assessment but were not excluded from analysis.

Twenty of the hips included in the data set had simultaneous femoral operations (femoral shortening ± derotational osteotomies) at the time of surgery. Five of the hips included in study had had previous surgery to either the femur or the acetabulum (four previous femoral varus producing derotational osteotomies and 1 previous shelf acetabulaplasty). Mean follow up time was 47 months (range 15 months to 92 months). In all but three hips there was improvement in femoral head containment on assessment of the post-operative radiographs. Radiographic data are summarized in Tables 1 and 3. Correlation coefficients displayed strong agreement in measurements for all parameters measured except Sharp’s angle which had moderate agreement.

**Table 1:** Radiographic outcomes following acetabular osteotomy.

	Pre-operative	Post-operative	Difference	*P* value
Lateral centre edge angle (degrees)	−2.1	39.2	41.3	<0.005
Sharp's angle (degrees)	49.9	34.575	−15.325	<0.005
Reimers migration index (% migration)	52.025	7.85	−44.175	<0.005
Tönnis angle (degrees)	23.55	5.525	−18.025	<0.005

**Table 2:** Patient demographics and diagnoses of patients included.

**Hip**	**Age**	**Sex**	**Side**	**Previous hip surgery**	**Diagnosis**	**GMFCS**	**Additional diagnoses**	**Follow up duration (months)**
1	18	F	L	Femoral osteotomy	CP	5	Microcephaly. Symptomatic focal epilepsy. Scoliosis	49
2	15	M	R	No	CP	3	Epilepsy	35
3	16	M	R	No	CP	3	Epilepsy, scoliosis	70
4	13	M	R	No	CP	3	N/A	65
5	19	**M**	**R**	No	CP	5	Epilepsy	64
6	19	M	L	No	CP	5	Epilepsy	62
7	16	M	L	Femoral osteotomy	CP	4	Epilepsy	31
8	12	F	R	No	CP	4	N/A	92
9	15	F	R	Femoral osteotomy	CP	3	N/A	39
10	16	M	R	No	CP	2	N/A	67
11	14	F	R	Femoral osteotomy	CP	3	N/A	42
12	14	F	R	No	Intracranial haemorrhage secondary to ITP^a^	2^c^	ITP	43
13	14	M	L	No	CP	3	N/A	84
14	12	F	L	No	PHH^b^	2^c^	Hydrocephalus, hyperthyroid	76
15	17	M	R	No	CP	1	N/A	33
16	18	M	L	No	CP	2	N/A	16
17	21	F	L	No	cp	1	N/A	63
18	32	F	L	Pelvic osteotomy (shelf)	cp	1	N/A	61
19	28	F	R	No	CP	2	N/A	21
20	15	F	L	No	CP	3	West syndrome	16
21	19	M	R	No	Cri Du Chat	5^c^	Cri du Chat, Aortic Stenosis, GORD, Sleep apnoea	17
22	18	M	L	No	Cri Du Chat	5^c^	Cri du Chat, Aortic Stenosis, GORD, Sleep apnoea	15
23	15	F	R	No	tetrapareseis post spinal cord infarction^d^	5^c^	Chiari malformation, SMA syndrome, scoliosis,	19

a ITP Immune thrombocytopenia (haemorrhage age 11),

b PHH = Post haemorrhagic Hydrocephalus (diagnosis age 5 months),

c Equivalent GMFCS.

d Diagnosis age 3.

**Table 3:** Changes in pre and post-operative radiographic measurements.

	L-CEA pre	L-CEA post	L-CEA difference	Sharp’s angle: Pre	Sharp’s angle: Post	Sharp’s Angle Difference	Reimer's index (% migration) Pre	Reimer's index (% migration): Post	Reimer's index (% migration): Difference	Tönnis angle: Pre	Tönnis angle Post	Tönnis angle Difference
Mean	−2.125	39.2	41.325	49.9	34.575	−15.325	52.025	7.85	−44.175	23.55	5.525	−18.025
Median	−1.75	41.75	38.25	50	34.5	−16.5	50.5	4.75	−41.75	25.5	8.5	−17
IQR	19.125	16.5	28.5	4.125	9.625	7.625	19	16.625	26.375	11.375	16.5	14.625

L-CEA (lateral centre edge angle)

On questioning in clinic 78% of patients or carers reported absence of pain at most recent follow up. None of the patients or their carers reported new onset of pain or worsening of their pain once initial post-operative pain subsided. Complications were seen in 12 patients summarized in Table 4. There was an average fall in haemoglobin levels of 32 g/L (pre-operative average 135 g/L post-operative average 102 g/L). Four patients required post-operative blood transfusions.

**Table 4:** Post-operative outcomes by patient.

Hip	Complications	Modified Clavien-Dindo Sink Complication Grade	Ongoing pain
1	Nil	0	N
2	Posterior non union – no treatment required	1	N
3	Nil	0	N
4	Nil	0	N
5	Recurrent subluxation with fixed flexion deformity, wound breakdown, arthritic change	3b	Y
6	Recurrent subluxation with fixed flexion deformity, arthritic change. Post operative blood transfusion	3b	Y
7	Post operative blood transfusion	1	N
8	Recurrent subluxation, post operative blood transfusion, symptomatic overcorrection	3b	Y
9	Femoral head avascular necrosis	3b	Y
10	Nil	0	N
11	Screw removal	3b	N
12	Transient lateral femoral cutaneous nerve palsy	1	Y
13	Nil	0	N
14	Nil	0	N
15	Post operative blood transfusion	1	N
16	Post operative blood transfusion	1	N
17	Inferior public rami stress fracture	1	N
18	Nil	0	N
19	Nil	0	N
20	Nil	0	N
21	Post operative blood transfusion	1	N
22	Nil	0	N
23	Post operative blood transfusion	1	N

## DISCUSSION

In this paper we present our experience of PAO in children and adults with hip subluxation or dislocation secondary to neuromuscular imbalance. The primary outcome of a stable and reduced hip was achieved in 87% of these challenging cases. There were statistically significant improvements in all radiographic parameters measured demonstrating an improvement in hip position and acetabular coverage of the femoral head. Patients in this cohort have comparatively deeper acetabulae, but often with severe posterior and lateral deficiency. The femoral deformity is complex, with anteverted femora, relative coxa valga, and retroflexion of the femoral head [23]. To achieve stability, careful consideration of the deformity is required, most easily visualized with CT imaging. Over correction is often required and accepted.

Management of patients with hip dysplasia and neuromuscular disease remains a complex surgical challenge. The natural history of hip dysplasia in the presence of muscle imbalance is of progressive subluxation and dislocation. At birth there is often little or no deformity present, and neuromuscular patients predominantly have primary abductor weakness. The unbalanced forces across the hip result in abnormal proximal femoral geometry, leading to hip instability and secondary acetabular dysplasia [3]. Untreated these patients often develop reduction in mobility, difficulty with nursing care, pain, and reduction in quality of life [24].

Different treatment options have been described to try and restore joint congruity or provide symptomatic relief in non-ambulatory patients, salvage procedures of the dislocated hip, with reported successful symptomatic improvement include femoral head resection and proximal femoral interposition arthroplasty. PAO preserves the native hip, prevents proximal hip migration and can preserve standing transfers. Some authors advocate for proximal femoral interposition arthroplasty in symptomatic patients who are GMFCS V [7, 25]. Patel *et al* published their outcomes of proximal femoral resection with soft tissue interposition in non-ambulatory patients with an average age of 22. Post-operatively they reported improved pain scores and sitting tolerance with a low complication rate and high patient satisfaction [25]. Others have had some success with prosthetic interposition arthroplasty. Silverio *et al* reported outcomes for use of arthroplasty in 12 Gross Motor Function Classification System (GMFCS) V patients (16 hips) with average age of 12 years. They demonstrated good to excellent outcomes in pain and range of movement in 44% of their patient cohort but with a revision rate of 18.7% [26].

**Graph 1:**
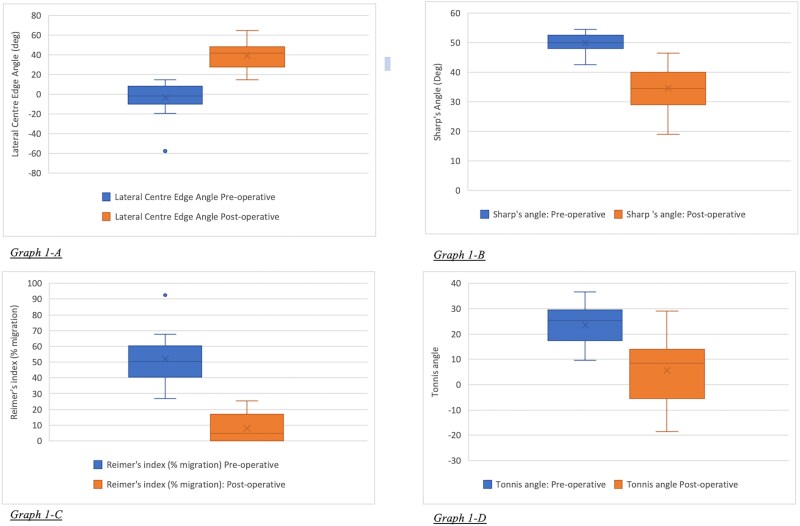
Changes in pre- and post-operative radiographic measurements. A) Lateral centre edge angle, B) Reimer’s Migration index, C) Sharp’s Angle, D) Tonnis angle.

Total hip arthroplasty has been also shown to be an effective treatment method for improve functional outcomes in small groups of appropriate neuromuscular pateints [27]. However, there are significant technical and patient factors which lead to an increased length of stay and post-operative infections (chest or urinary) or peri-prosthetic fractures with revision rates up to 35.3% [27–30]. Where femoral head deformity and chondral loss exceeds hip preservation, PAO may not be appropriate, but we have found good symptomatic relief in cases with chronic dislocation despite moderate deformity. The optimal choice of procedure needs careful Multi-Disiplinary Team (MDT) discussion involving care givers, therapists and patients where appropriate.

The outcomes following PAO in our study are comparable to others published in the literature. Miller *et al* reported management of 18 hips in skeletally mature patients with PAO, although less severely affected than our cohort. They demonstrated comparable improvements in radiological measurements on post-operative radiographs (post op Tönnis angle 6.5, L-CEA 31, Reimer's index 21%). There were similar levels of complications seen post-operatively (39%), reflective of the patient group [31]. Chen *et al*, looked at the management of 21 hips with dysplasia secondary to CP, treated with PAO with or without concordant femoral osteotomy. They found a higher re-subluxation rate of 30% [32].

Incomplete pelvic osteotomies, combined with osteotomy of the femur, are frequently used in the management of neuromuscular patients. The Dega osteotomy has long been utilized in the treatment of spastic hip dysplasia particularly in younger patient groups. The preserved plasticity of the ileum, particularly in non-ambulant children, means the upper age limit of these osteotomies can be extended in neuromuscular disease. In a review of 33 subluxed or dislocated hips in skeletally mature patients treated by incomplete transiliac osteotomy, comparable improvement in radiologic measurements and pain to that of our study has been reported [33]. Whilst the complication profile of the PAO should not be ignored, the procedure allows more powerful correction with complete reorientation of the acetabular fragment in all planes. Acetabular version is addressed whilst simultaneously achieving improvement in posterior and lateral coverage, a useful tool in the presence of severe dysplasia and the posterior deficiencies often seen in this group. Attempting to address axial plane deformities through the femur alone can be difficult to achieve.

As would be expected, given the increased surgical complexity of the operation, and decreased pre-operative functional status of the patients, complication rates are higher than compared to treatment of hip dysplasia without neurological disease [34]. About 87% of patients required concordant femoral shortening operations to allow for reduction of the hip at the time of the PAO, again reflecting the increased complexity of the surgery in the presence of neuromuscular disease.

One important consideration is the prolonged rehabilitation that is required following hip reconstruction in patients with neuromuscular disorder. Anecdotally this can be up to 2 years for ambulant patients. We are often referred these patients as they reach adolescence, and we know that children with cerebral palsy decline in gross motor function as they move into young adulthood. The change in power to weight ratio impacts mobility, particularly affecting patients GMFCS III and IV. The need for hip reconstruction in the presence of severe hip migration may coincide with this critical age impacting post-operative mobility status and young people and their families must be counselled appropriately*.*

We recognise that this paper has limitations including those inherent with retrospective data collection. There was no formal standardization of the pre- and post-operative radiographs. This combined with the difficulties in performing adequate pelvic radiographs in patients who may have contractures or spinal deformity, may cause error in the calculation of the radiographic data. Patient reported outcome measures (PROMs) data collection in this patient group are difficult. In our series, we have only used clinical and radiological outcomes and not collected formal PROMs data. From review of the case notes, at clinical follow up all patients who did not have a recurrence (three hips), reported improved pain, mobility, and ease of transfer but we recognise this is a crude measurement, and going forward we need to be collecting outcome measures relevant to the patients and their carers. Study numbers remain relatively small and there is heterogeneity in pathology and disability amongst the patient group. The mean follow-up for these patients was 47 months which represents short term outcomes and further work is required to assess the medium to long term outcomes in these patients. Additionally due to the complex nature of this patient cohort, with variations in severity of hip pathology and other co-morbidities, it is difficult to perform matched cohort studies to assess symptomatic improvement. Nevertheless, we feel the use of PAO for these patients has transformed our ability to deal with complex hip deformity in a challenging patient group, with what appears to be satisfactory results.

## CONCLUSION

In this paper we present our outcomes and experience of the Bernese PAO in skeletally mature patients with neuromuscular disorders. Management of these patients remains a difficult challenge with complexities in both the operative and post-operative period. Our experience has shown that the Bernese PAO is a suitable operation for management of subluxing hips in patients with neuromuscular disease.

## EXAMPLE CASES

### Case 1

**Figure 1:**
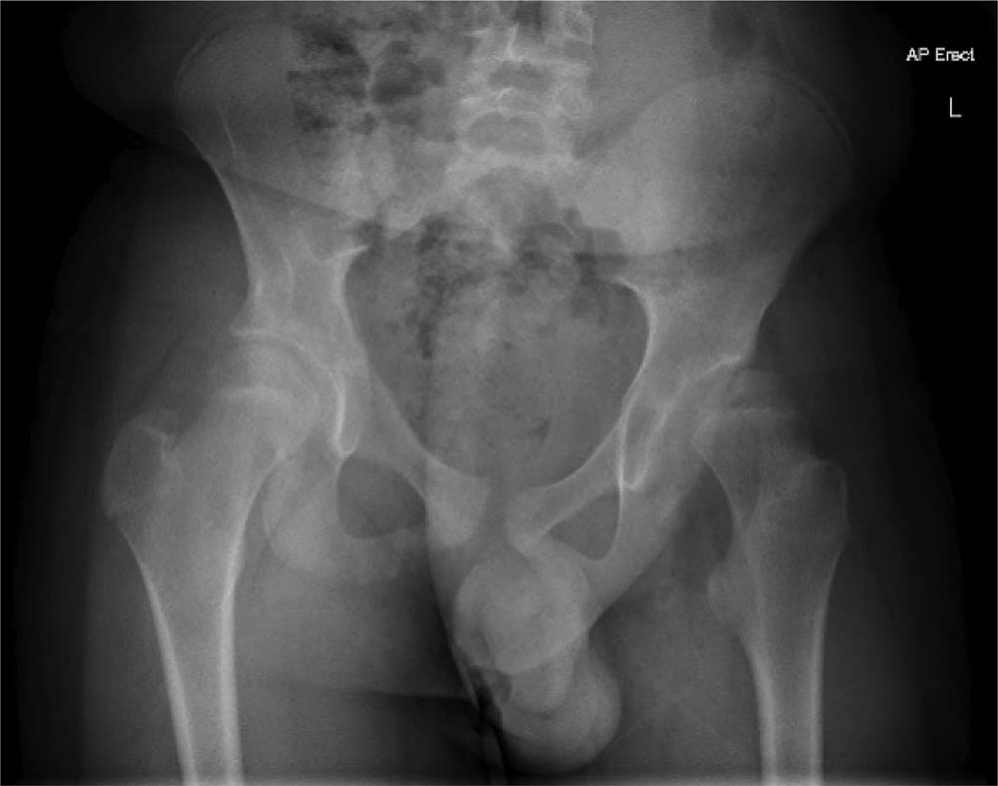
Case 1 pre-operative radiograph.

**Figure 2:**
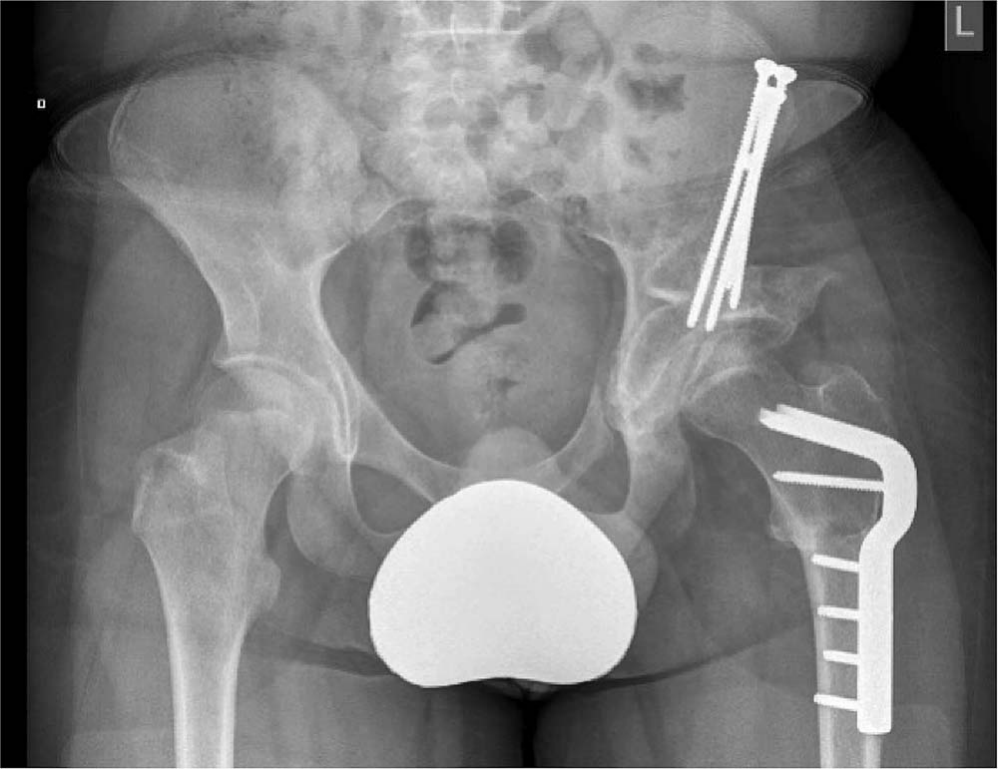
Case 1 post-operative radiograph.

Pre-operative (Fig. 1) and post-operative (Fig. 2) radiographs for a GMFCS III patient treated with peri-acetabular and femoral osteotomy. In this case the bone graft taken from the femoral shortening has been utilized to aid correction in the pelvis. Post-operatively this patient and their care givers reported reduced pain and subjective improvements in mobilising with aids.

### Case 2

**Figure 3:**
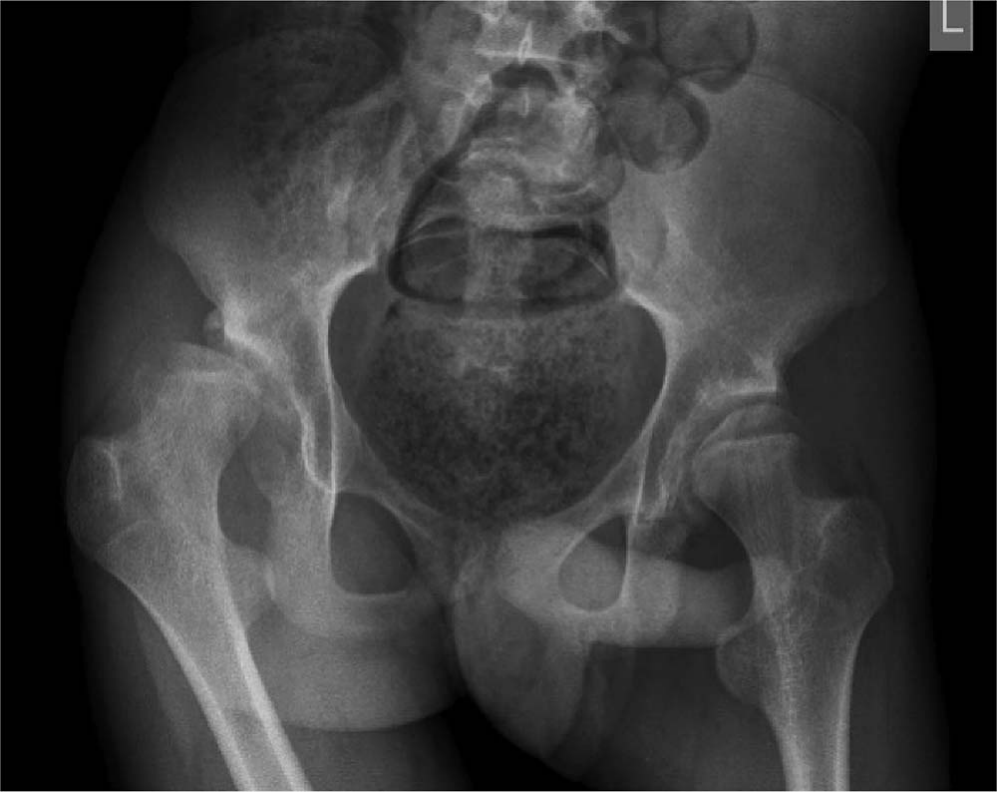
Case 2 pre-operative radiograph.

**Figure 4:**
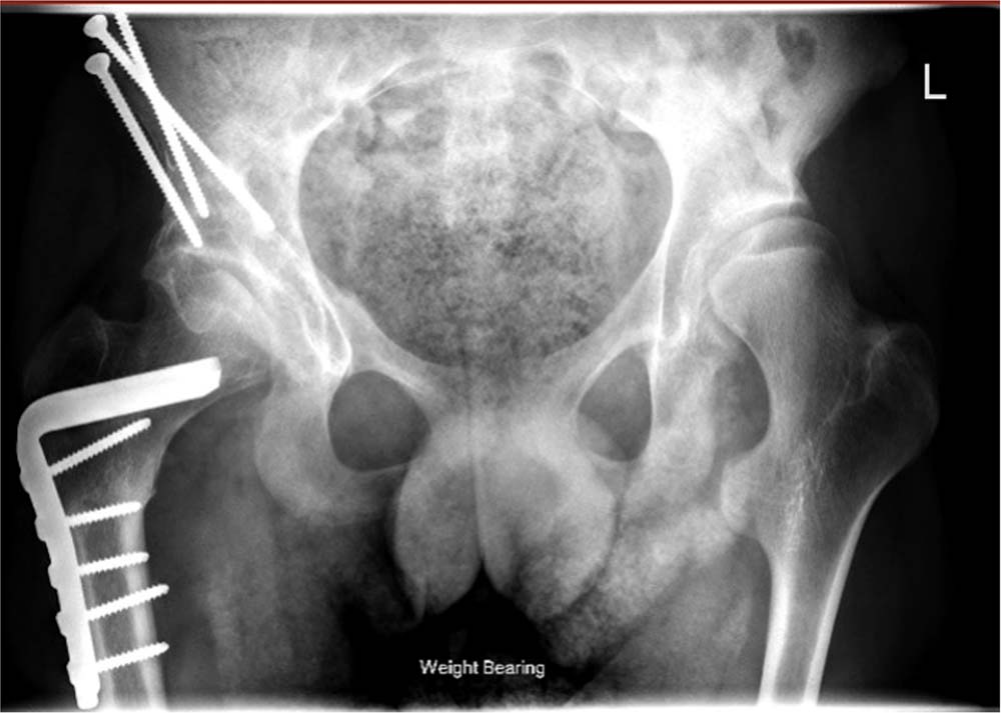
Case 2 post-operative radiograph.

Pre-operative (Fig. 3) and post-operative (Fig. 4) radiographs for a GMFCS IV patient treated with peri-acetabular and femoral osteotomy. In this patient there is ‘squaring off’ of the femoral head due to the deforming forces from overlying spastic hip abductors and the tight superior capsule, as well as post-operative hypertrophied and elongated LT producing a triangulation effect. The family are counselled pre-operatively about the risk of post operative pain in an incongruent joint. However, our experience is that non-ambulatory patients do not seem to have the pain expected and have not gone on to head -neck resection.

## Data Availability

The data underlying this article will be shared on reasonable request to the corresponding author.
